# The effect of induced blur on the Beery-Buktenica developmental test of visual-motor integration and its supplemental tests

**DOI:** 10.1371/journal.pone.0237807

**Published:** 2020-08-20

**Authors:** Rebecca Findlay, Joanna Black, Bert van der Werf, Carol Chelimo, Cameron C. Grant, Nicola Anstice

**Affiliations:** 1 School of Optometry and Vision Science, University of Auckland, Auckland, New Zealand; 2 School of Population Health, University of Auckland, Auckland, New Zealand; 3 Department of Paediatrics—Child and Youth Health, School of Medicine, University of Auckland, Auckland, New Zealand; 4 Discipline of Optometry and Vision Science, University of Canberra, Canberra, Australia; The University of Melbourne, AUSTRALIA

## Abstract

**Background:**

The Beery-Buktenica Test of Visual-Motor Integration (Beery VMI) is a commonly used standardized test of visual-motor integration. Performance on the test is related to academic achievement, but the effect of reduced visual acuity on test results is unknown. This study determined the visual acuity demand and the spacing of the test forms for the Beery VMI and its supplemental tests and investigated the effect of induced optical blur on test results in both adults and children.

**Methods:**

The overall size and critical detail size of each form and the distance between the center of each form and its adjacent crowding source were measured. The visual acuity demand and angular separation of forms were calculated. Two groups of participants (adults aged ≥18 years [n = 19] and children aged 7–12 years [n = 20]) completed four sessions in which they performed the Beery VMI and the supplemental tests under different blur conditions (habitual near correction, monocular spherical blur, binocular spherical blur and binocular astigmatic blur) in a randomized order.

**Results:**

Stroke width, overall form size and box size remained constant throughout the Beery VMI, whereas these reduced with increasing difficulty for the supplemental tests. Reduced near visual acuity from simulated blur resulted in reduced mean scores for the Beery VMI and its supplemental tests, compared with habitual near vision in both adults and children. Binocular spherical blur had the most detrimental effect (p<0.001), followed by binocular astigmatic blur (p<0.001) then monocular spherical blur (p = 0.022).

**Conclusions:**

In individuals with uncorrected spherical or astigmatic ametropia, reduced scores on the Beery VMI and its supplemental tests may be due to impaired near visual acuity and not reflect reduced visual-motor abilities. This highlights the importance of excluding reduced near visual acuity as a cause of reduced performance before diagnosing impairment and initiating treatment strategies for visual-motor integration.

## Introduction

The Beery-Buktenica Test of Visual-Motor Integration (Beery VMI) is a commonly used, standardized test of VMI. However, its administration can be problematic in children with undiagnosed vision disorders because the effect of reduced near visual acuity (VA) on test results has not been systematically explored. The Beery VMI involves copying a series of geometric forms of increasing difficulty [1]. It is commonly used by neuropsychologists and occupational therapists [2, 3] as it has been standardised multiple times on more than 13,000 children and 1,000 adults for both individual and group administration [1]. It has high reliability and intra-scorer and inter-scorer agreement in adults and children [1, 4–6].

The Beery VMI includes supplemental tests of Visual Perception (VP) and Motor Coordination (MC). The VP supplemental test assesses visual analysis skills in a format which requires minimal motor input. A reference form is shown with several similar shapes below it; the subject must choose which shape is identical to the original form. The MC test minimises visual analysis by providing examples, starting dots, and paths as visual guides for the required motor tasks. For each item on the MC supplemental test, the participant is asked to draw within specific lines on the form. To limit the size and cost of the testing booklets, the size of the items on the VP and MC are different to those on the VMI [1], and the size of test forms decreases with increasing difficulty in these supplemental tests. Although the accompanying instruction manual advises examiners to refer any patient suspected of having reduced VA for a vision examination, there is no specific information regarding the minimum required VA, illumination or testing distance.

Several studies have shown that the performance of children on the Beery VMI is related to academic achievement [7–9] and VMI is a strong predictor of academic success in children at ages 5–8 years [10–12]. VP and VMI scores are significantly associated with reading achievement, handwriting skills and written mathematics ability in school-aged children [13–17]. Likewise, visual perceptual dysfunction is significantly more prevalent in 6–12 year old children with learning difficulties than those without [18].

Uncorrected ametropia is associated with poorer performance on visuocognitive and visuomotor tests [19] and impaired visual perceptual skills [20], but data on the relationship between refractive error and VMI is limited. Some studies have found significantly lower VMI or VP supplemental test scores in children with uncorrected hyperopia associated with reduced binocular near VA or poor stereopsis [21], bilateral uncorrected astigmatism [22] and uncorrected hyperopia and astigmatism [23]. In contrast, other studies have shown no association between VMI scores and refractive error [24]. These differences may be the result of differing refractive error definitions and the variable effect of refractive error, particularly hyperopia and astigmatism, on VA. It is unclear from the current literature whether reduced performance on the VMI and its supplemental tests is due to reduced VA or to delayed development of visual perceptual skills in children with refractive error, or a combination of both of these factors.

Therefore, the purpose of this study was to determine the VA demand and the spacing of the test forms of the Beery VMI and its supplemental tests, in order to investigate the effect of reduced near VA (to a predetermined acuity level) caused by induced optical blur on the results of these tests in paediatric and adult participants.

## Methods

Ethics approval was obtained from the University of Auckland Human Participants Ethics Committee and the research followed the tenets of the Declaration of Helsinki. Adult participants and parents of child participants provided written informed consent, with children providing written assent.

### Test design

The overall size of each test form (test shape) along its longest dimension, critical detail size for each test form (smallest detail of form), and the distance between the center of each form and an adjacent source of crowding were measured using Vernier callipers (Table 1). The VA demand was calculated based on the critical detail size and a viewing distance of 40 cm. The angular separation between each form and its adjacent crowding source was then calculated.

**Table 1:** Features considered to be critical details and crowding sources for the Visual Motor Integration, Visual Perception and Motor Coordination tests.

Test		Critical detail	Crowding source
Visual Motor Integration		Stroke width of test forms	Adjacent form
Visual Perception		Stroke width of reference forms	Box surrounding reference form
Motor Coordination	Forms 4–21	Diameter of starting dots	Adjacent form
	Forms 22–30	Width of border lines of test forms	Adjacent form

### Participants

Two groups of participants, adults aged 18 years or older and children aged 7–12 years, were recruited via convenience sampling. Participants were excluded from the study if they had habitual near VA less than 0.1 logMAR in either eye, a difference in VA between their eyes of greater than 0.1 logMAR, or any self-reported pre-existing ocular health or neurological conditions. All participants were assessed (by the lead author) at the University of Auckland, School of Optometry and Vision Science or in their own homes.

### Test procedures

The participants’ right, left and then binocular habitual near VA were measured with the Sloan Letter near VA chart (Good-Lite Company) viewed at 40 cm. With their habitual near prescription in a trial frame, participants were asked to view the 0.3 logMAR line on the near VA chart. With the left eye occluded, plus spherical lenses were added in front of the right eye until the 0.3 logMAR line was no longer visible. The last lens where the 0.3 logMAR line was visible was recorded. This procedure was repeated for the left eye then binocularly. To induce with-the-rule astigmatic blur, the assessor added negative cylinders with the axis vertical and a balancing plus spherical lens of half the cylindrical power to maintain a plano spherical equivalent.

Participants completed four sessions, in which they performed the Beery VMI and the VP and MC supplemental tests under the different blur conditions (habitual near correction, monocular spherical blur (right eye), binocular spherical blur and binocular astigmatic blur) in a randomized order. The interval between sessions ranged between one and 27 weeks.

The Beery VMI was performed according to the test directions given in the manual [1], with the exception that participants were asked to perform the test at 40 cm, to ensure the level of blur and visual angle of the test forms remained constant. The VMI assessment was followed by the VP and MC supplementary tests for all participants at each session. Forms were scored by the lead author, while blinded to the blur condition, according to standardized criteria [1].

### Statistical analysis

A sample size of 14 participants was estimated to be sufficient to detect a 15 point difference in the VMI standardized score (one standard deviation [SD] away from the mean), with a power of 80% and a two-sided significance of 5%, assuming a standard deviation of 13.7 [23]. This estimate was increased to 20 participants to allow for a predicted dropout of 30% for the four measurement visits combined.

Raw scores of the VMI and the VP and MC supplemental tests were converted to standard scores (mean of 100, standard deviation of 15) using the tables provided in the manual [1]. Scores were fitted simultaneously using a full linear mixed model including all interactions between the independent variables blur condition, test type (VMI or VP or MC supplementary test), sex, age group (child or adult) and age (years). Previous experimental test condition and test order were included to account for the repeated measurements and possible learning effects. The model also included participant identifier and date of measurement as random factors. The best model was that with the minimum value for the Akaike Information Criterion (AIC) [25]. All variables were included in the initial model and then those that decreased the AIC value were removed from the model; this was also done for the random terms. Data analysis was conducted using R (version 3.5.1) [26]. The models were fitted with the lmer function from the lme4 package of R [27].

## Results

### Test design

For the Beery VMI, stroke width, overall test stimulus size and box size remained constant for all forms throughout the test. For both the VP and MC supplemental tests, overall test stimulus size, box size and inter-stimulus distance reduced with increasing difficulty (Tables 2 and 3).

**Table 2:** Measurements of form size, critical detail and visual acuity demand for the Visual Motor Integration, Visual Perception, Motor Coordination tests.

Test	Form	Overall form size (mm)	Critical detail size (mm)	Visual acuity demand of critical detail at 40 cm (logMAR [MAR])
VMI	All Forms	50.6	1.3	1.04 (11’)
VP	Forms 4–9	20.0	0.7	0.78 (6’)
	Forms 10–16	17.5	0.5	0.6 (4’)
	Forms 17–30	10.0	0.3	0.3 (2’)
MC	Forms 7–11	51.4*	4.0	1.53 (34’)
	Forms 12–16	44.8*	2.5	1.30 (20’)
	Forms 17–21	46.0*	1.7	1.15 (14’)
	Forms 22–27	39.4*	0.3	0.3 (2’)
	Forms 28–30	27.0*	0.3	0.3 (2’)

VMI: Visual Motor Integration, VP: Visual Perception, MC: Motor Coordination.

* Forms in these groups were not of uniform size, mean sizes reported.

**Table 3:** Measurements of form size and distance between form size and crowding detail for the Visual Motor Integration, Visual Perception, Motor Coordination tests.

Test	Form	Overall form size (mm)	Center to center separation of forms and crowding detail (mm)	Angular separation of forms and crowding detail at 40 cm (degrees)
VMI	All Forms	50.6	84.2	12.0
VP	Forms 4–9	20.0	15.5	2.2
	Forms 10–16	17.5	12.5	1.8
	Forms 17–30	10.0	6.0	0.9
MC	Forms 7–11	51.4*	56.7^†^	8.1
	Forms 12–16	44.8*	55.7^†^	7.9
	Forms 17–21	46.0*	55.3^†^	7.9
	Forms 22–27	39.4*	45.2^†^	6.4
	Forms 28–30	27.0*	48.8^†^	7.0

VMI Visual Motor Integration, VP Visual Perception, MC Motor Coordination.

* Forms in these groups were not of uniform size, mean sizes reported.

† Center to center form separations in these groups were not uniform, mean separations reported.

### Experimental results

**Fig 1:**
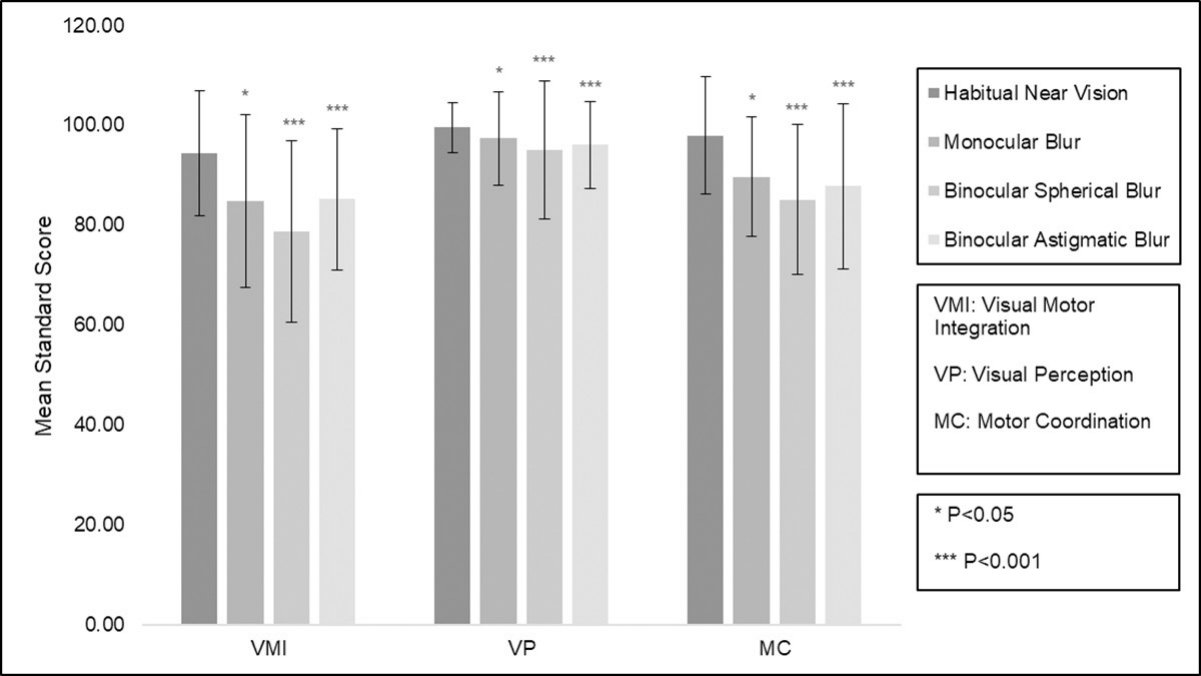
Mean (± standard deviation) standardised scores for Visual Motor Integration and supplemental tests for each blur condition in children (n = 20).

**Fig 2:**
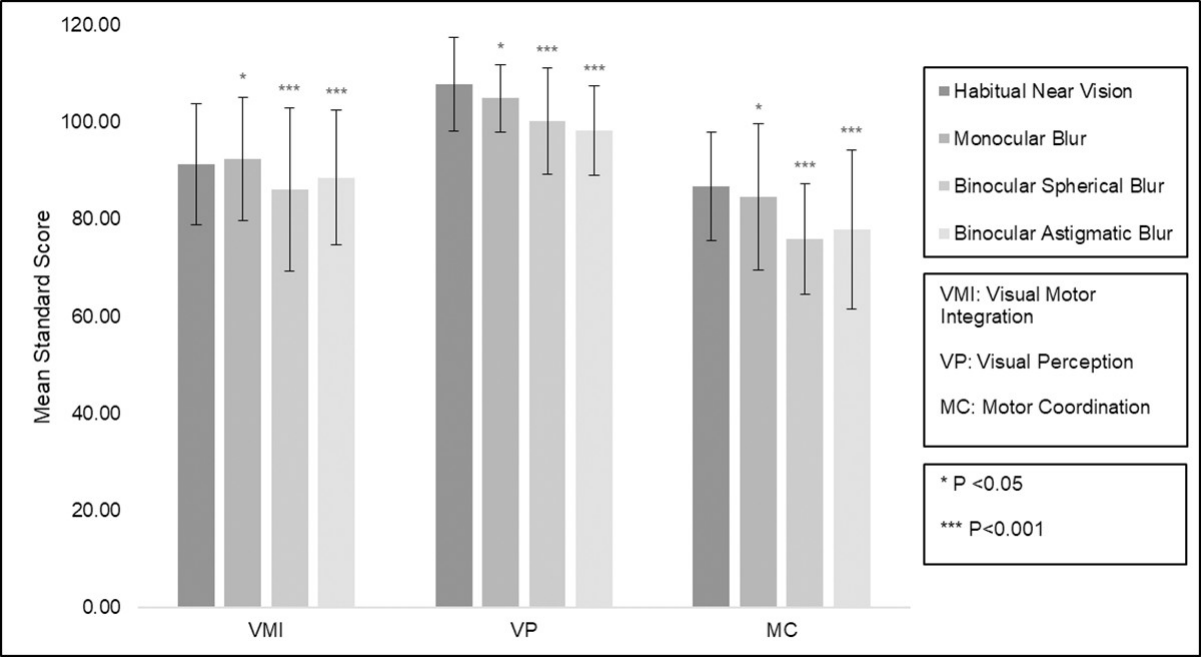
Mean (± standard deviation) standardised scores for Visual Motor Integration and supplemental tests for each blur condition in adults (n = 19).

Twenty children (mean [SD] age of 9.8 [1.9] years, 60% female) and nineteen adults (mean [SD] age of 37.3 [6.4] years, 63% female) participated in this study. Mean (SD) of binocular near VA was -0.07 (0.10) logMAR for children and -0.09 (0.08) logMAR for adults. Mean levels of optical blur required to give VA of 0.3 logMAR are shown in Table 4. All participants were able to complete the VMI and both supplemental tests under all conditions of defocus. Mean standard scores (SD), with and without induced optical blur, are shown in Figs 1 and 2.

**Table 4:** Mean optical blur required to reduce near visual acuity to 0.3 logMAR.

Group	Monocular Spherical Blur (D) Mean ± SD	Binocular Spherical Blur (D) Mean ± SD	Binocular Astigmatic Blur (DC) Mean ± SD
Children (n = 20)	2.89 ± 0.54	3.00 ± 0.69	2.10 ± 0.77
Adults (n = 19)	2.45 ± 0.69	2.58 ± 0.56	2.00 ± 0.58

D = Diopters DC = Diopters Cylinder.

### Analysis

Baseline VMI, VP and MC mean scores were within the age-appropriate standardized normal range for both adults and children. Linear mixed model analysis showed that reduced near VA from each of the simulated refractive blur conditions was associated with reduced mean scores for each of the VMI, VP and MC tests compared with habitual near vision. For each of the tests, the greatest decrease in mean score was due to binocular spherical blur (-9.63 points, t = -5.94, p<0.001), followed by binocular astigmatic blur (-6.91 points, t = -4.15, p<0.001) and then monocular spherical blur (-3.68 points, t = -2.29, p = 0.022). Age, previous experimental test condition and test order were removed from the maximal model as their inclusion resulted in higher AIC values. While standard scores were significantly reduced for children compared with adults (-7.79 points, t = -4.19, p<0.001) and males compared with females (-3.96 points, t = -2.65, p = 0.008), the effect of induced blur was the same for all groups.

## Discussion

Reduced near VA from induced optical blur was associated with poorer performance on the Beery VMI and its VP and MC supplemental tests for both children and adults. Our findings suggest that in individuals with uncorrected spherical or astigmatic ametropia, particularly if it is binocular, reduced scores on the Beery VMI and its supplemental tests may be the result of reduced near VA and not reflect reduced visual-motor abilities.

The findings from this study support previous studies which have shown a reduction in VMI scores in children with hyperopia and reduced near visual function, children with bilateral uncorrected astigmatism and those with hyperopia and astigmatism [21–23]. The participants in this study required an average of 3.00 D of spherical blur and 2.00 D of astigmatic blur to achieve near VA of 0.3 logMAR. Harvey *et al*. [22] found that both astigmatism (≥1.00 DC) and reduced near VA were significantly associated with performance on the VMI and the VP supplemental test. Similarly, Kulp *et al*. [21] found reduced VMI and VP scores in children with hyperopia (≥3 D and ≤6 D) and reduced near VA but no difference between emmetropic children and children with hyperopia overall; their findings suggest that deficits in near VA associated with optical blur were likely the cause of the reduction in performance rather than the presence of refractive error alone. In contrast, Roch-Levecq *et al*. [23] found reduced test scores in children with ametropia but did not find any correlation between VMI scores and either distance or near VA.

Uncorrected refractive error, in particular uncorrected hyperopia and astigmatism, may result in reduced near VA [28–30]. Studies of near VA in typically developing children are limited. However, up to 5% of children may have near VA of 0.3 logMAR or worse and children who are born preterm may have poorer near VA than children born full-term [31, 32]. Additionally, adults with presbyopia will have reduced near VA unless appropriately corrected for their near working distance. An acuity reserve, whereby the print size of reading materials is double the just-readable print size (near VA), is recommended for comfortable reading [33].

For the VP and MC supplemental tests, overall form size reduces with increasing form difficulty. Likewise, there is a reduction in the critical detail size and consequently the visual angle, increasing VA demand as patients progress through the test. In the present study, forms 17–30 of the VP supplemental test and 22–30 of the MC supplemental test were at the limit of the participants’ near VA under induced blur conditions. Therefore, the absence of an acuity reserve may have affected performance on these tasks. Compared to the VMI test, an individual with inadequate near VA may perform more poorly on the VP and MC supplemental tests due to insufficient VA to resolve the critical detail of the test forms rather than an actual deficiency in visual perception or motor coordination.

The VA demand remains constant throughout the test for the Beery VMI due to standard form and critical detail size which remain unchanged throughout the test. Even considering an acuity reserve, this test should be accessible to individuals with near VA of 1.34 logMAR or better; however, VMI performance in our study was decreased with induced optical blur sufficient to reduce near VA to only 0.3 logMAR. This is in agreement with studies of children with uncorrected astigmatism and hyperopia with reduced near visual function which also found reduced performance on the VMI despite the supra-threshold size of the test forms [21, 22]. Our findings suggest that the induced blur affects not only VA but also visual-motor integration. Because optical blur was introduced for only short time periods in emmetropic children and children with corrected ametropia, our results suggest that the reduction in VMI scores seen with optical defocus in this study were due to a direct effect of the induced blur rather than deficient visual-motor development.

Visual perceptual skills have been associated with academic ability [13–15] and are often investigated in children with academic delays. Additionally, deficient visual perceptual skills have been shown to improve with therapy [34–36]. Recommending a vision examination for individuals who “display behavior that causes an examiner to suspect a visual acuity problem” [1], as suggested in the manual, appears to be insufficient to rule out reduced VA associated with optical blur, given that decreased performance on the VMI and its supplemental tests may be the only sign of reduced VA for an examiner. Therefore, children should receive a comprehensive eye examination and correction of refractive errors that reduce near VA prior to VMI testing. Where VMI testing is used for screening in a group setting, children with reduced VMI scores should be referred for a comprehensive eye examination prior to the diagnosis of a deficit in VMI and initiation of therapy.

Participants were asked to perform the test at 40 cm to maintain consistent blur and visual angle of the test stimuli. These instructions differ from those in the manual which does not specify a test distance [1], allowing the individual to perform the test at a self-determined near distance allowing for stature and comfortable viewing distance. This can reduce the VA demand, particularly in children who may have shorter working distances; however, our results suggest that near blur affects performance even where the test form is well above the VA threshold.

Crowding refers to the detrimental influence of an object’s surroundings on visual discrimination [37]. Crowding is important in individuals with macular degeneration, amblyopia and dyslexia and is commonly measured by considering the distance between the centers of adjacent targets [37]. In addition to the reduction in the form size for the VP and MC supplementary tests, there is also a reduction in the distance between the center of forms and their crowding detail as form difficulty increases, particularly for the VP supplementary test. Participants in this study were excluded if they had any self-reported eye disease or neurological condition, or a difference in VA between the eyes, thus, crowding is unlikely to have had an effect in this study. However, in individuals with amblyopia, macular degeneration or dyslexia, crowding may result in impaired performance on the VP supplemental test due to reduced spacing of forms in this test [37].

This study has several potential limitations. Participants wore their habitual near correction and did not receive a full subjective refraction before VMI testing. Thus, participants may not have been optimally corrected, and this may have affected their performance. However, participants still performed significantly better with their habitual correction than with induced blur. Participants repeated VMI testing on multiple occasions, at different inter-test intervals. Repeat testing may have been subject to learning effects and the impact of differing inter-test intervals on the results is unknown, however, test order was randomised to minimise the influence of these factors on the results. Additionally, although induced blur is a method commonly used in research [28, 29, 38], it differs from uncorrected refractive error and participants with induced blur may perform differently to those with uncorrected refractive error to which they have adapted. In this study, we used different levels of optical blur for different participants to achieve a standard near VA level, rather than a constant level of optical blur. Inducing blur to give a pre-determined VA level allowed us to examine the effect of reduced VA on VMI results rather than merely refractive error as previous studies have shown conflicting results. However, this meant each participant experienced different levels of optical blur for a given test condition, and our study design does not allow us to provide details of minimum blur thresholds for performing the VMI test.

Testing in this study was undertaken under daylight luminance conditions. VA is detrimentally affected by low luminance and contrast conditions and larger critical detail size is required to give the same VA in these conditions. While in-office administration of the Beery VMI is likely to be performed in high luminance conditions, group administration of the test may be conducted in classroom settings where illumination levels can be variable and may not meet minimum standards [39]. The Beery VMI manual does not provide guidelines for illumination and further study is required to determine the effect of luminance conditions on performance on the Beery VMI and its supplemental tests, and the minimum luminance requirements for effective administration.

In summary, our findings highlight the importance of excluding reduced near VA due to optical defocus as the cause of reduced performance on the Beery VMI and its supplemental tests before diagnosing impairment and initiating treatment strategies. While test forms on the VP and MC supplemental tests were at the limits of near VA with induced blur in our study, performance on the Beery VMI was also reduced despite forms being within the VA threshold. A comprehensive vision examination and correction of any refractive error that reduces near VA should be completed before VMI assessment, and children who have reduced scores following assessment of VMI should receive a vision examination prior to diagnosis and treatment.

## Supporting information

S1 Dataset (XLSX)
